# Treatment of corticosteroid refractory immune checkpoint inhibitor myocarditis with Infliximab: a case series

**DOI:** 10.1186/s40959-021-00095-x

**Published:** 2021-03-30

**Authors:** Robert S. Zhang, Allison Padegimas, Kathleen M. Murphy, Peter T. Evans, Carli J. Peters, Christopher M. Domenico, Mahesh K. Vidula, Paul J. Mather, Marisa Cevasco, Roger B. Cohen, Joseph R. Carver, Rupal P. O’Quinn

**Affiliations:** 1 Department of Medicine, University of Pennsylvania, PA Philadelphia, USA; 2 Division of Cardiovascular Medicine, University of Pennsylvania, PA Philadelphia, USA; 3 Division of Infectious Disease, University of Pennsylvania, PA Philadelphia, USA; 4 Department of Pharmacy, University of Pennsylvania, PA Philadelphia, USA; 5 Department of Cardiothoracic Surgery, University of Pennsylvania, PA Philadelphia, USA; 6 Division of Hematology-Oncology, University of Pennsylvania, PA Philadelphia, USA

**Keywords:** Immune checkpoint inhibitor, Myocarditis, Infliximab, Cardio‐oncology, Pembrolizumab, Nivolumab

## Abstract

**Background:**

Glucocorticoid treatment remains the cornerstone of therapy for immune checkpoint inhibitor (ICI) myocarditis, but data supporting the use of additional immunotherapy for steroid refractory cases remains limited. We investigate the safety and efficacy of infliximab in patients with ICI myocarditis who are refractory to corticosteroids. Additionally, we highlight the importance of a multi-disciplinary approach in the care for these complex patients.

**Methods:**

We retrospectively identified consecutive patients who developed ICI myocarditis at our institution between January 2017 and January 2020. Baseline characteristics, laboratory data and clinical outcomes were compared between patients who received infliximab and those who did not.

**Results:**

Of a total of 11 patients who developed ICI myocarditis, 4 were treated with infliximab. Aside from age, there were no significant differences in baseline patient characteristics between the two groups including total number of ICI doses received and duration from initial ICI dose to onset of symptoms. The time to troponin normalization was 58 vs. 151.5 days (*p* = 0.25). The duration of prednisone taper was longer in the infliximab group (90 vs. 150 days *p* = 0.32). All patients survived initial hospital admission. Over a median follow-up period of 287 days, two of the 4 patients died from sepsis 2 and 3 months after initial treatment of their myocarditis; one of these patients was on a steroid taper and the other patient had just completed a steroid taper.

**Conclusions:**

Infliximab, despite its black box warning in patients with heart failure, may be a safe and effective treatment for ICI myocarditis.

## Background

Immune checkpoint inhibitors (ICIs) have brought about a paradigm shift in the treatment of many cancers. ICIs enhance the immune system’s detection and targeting of tumor cells and improve progression-free and overall survival in a growing number of adult cancers that are refractory to traditional chemotherapeutic agents [1, 2]. With increasing use of ICIs, immune-related adverse events (irAEs) have become more prevalent, and most commonly include endocrinopathies, pneumonitis, colitis and hepatitis [3]. Immune-related adverse events are more severe and more likely to occur with combinations of checkpoint inhibitors [4–6]^.^ While cardiovascular toxicity is less frequent than other irAEs with a reported incidence of 1.14 %, it is associated with a high mortality rate of 25–50 % [5, 7–9]; making it unsurprising that the number of cases published discussing life-threatening cardiotoxicity continues to rise [10]. There are various proposed treatment algorithms for ICI-mediated cardiotoxicity but standardized guidelines are lacking. While glucocorticoid therapy has become a widely accepted treatment, only about 50 % of patients with fulminant myocarditis respond to glucocorticoid monotherapy [11]. Various additional immunosuppressive agents have been used in steroid-refractory cases; however, data supporting the use of these therapies are limited. In this retrospective study, we describe our single center experience with four patients who had steroid-refractory ICI myocarditis that improved with infliximab administration. We compare their clinical characteristics and outcomes with those who did not require immunosuppression beyond steroids. Finally, we provide a suggested treatment algorithm created by a multi-disciplinary team of experts in cardio-oncology, heart failure, infectious disease, medical oncology and cardiothoracic surgery.

## Methods

### Study population and data collection

We retrospectively identified consecutive patients who developed ICI myocarditis at our institution between January 2017 and January 2020. ICI myocarditis was diagnosed in consultation with a cardio-oncology expert based on standard histological features present on endomyocardial biopsy or a guideline-recommended scoring system incorporating several factors including clinical presentation, biomarkers and imaging features [12]. Grading of ICI myocarditis was performed according to the American Society of Clinical Oncology (ASCO) practice guidelines [13]. Patients treated with infliximab were those with persistent evidence of cardiac dysfunction such as malignant arrhythmias or cardiogenic shock despite treatment with high doses of glucocorticoids [14]. Clinical data including baseline demographics, medications, laboratory values, and the results of echocardiography, cardiac MRI (cMRI) and endomyocardial biopsy were obtained from electronic medical records abstracted by a physician. This study was approved by the University of Pennsylvania Institutional Review Board.

### Covariates

Covariates were selected based on clinical relevance and prior studies [7]. Demographic covariates included age, sex, and race. Clinical covariates included body mass index (BMI), baseline cardiovascular risk factors, baseline echocardiographic data if available, and medications. Cancer-specific covariates included type of cancer, the type and number of cycles of ICI treatments received, and any exposure to prior cardiotoxic chemotherapy and/or chest radiation. Myocarditis-specific covariates included clinical presentation, ischemic cardiac evaluation, initial and peak cardiac biomarkers (i.e., troponin), time to resolution of cardiac biomarkers, treatment regimen for myocarditis and if available, cardiac MRI and endomyocardial biopsy results. Admission troponin was defined as first serum troponin measured, and peak troponin was the highest measured troponin value. Time to troponin resolution was defined as the time (in months) from first troponin elevation to the first troponin that was undetectable.

### Outcomes

The outcomes of interest were major adverse cardiac events (MACE), which we defined as cardiovascular death, cardiac arrest, cardiogenic shock, hemodynamically significant heart block and hemodynamically significant arrhythmias.

### Statistical analysis

Continuous variables are presented as mean ± standard deviation (SD) or median with interquartile range (IQR) for skewed data. Categorical data are expressed as frequencies and proportions. Continuous variables were compared using unpaired Student’s t-test for normally distributed continuous variables, the Wilcox rank-sum when normal distributions were not met, and Fisher’s exact test for categorical variables. Analyses were performed using Stata software (version 15.0; StataCorp LP, College Station, TX). All analyses were two-sided and a *p* value less than 0.05 was considered significant.

## Results

Of a total of 11 patients at our institution who developed ICI myocarditis, 4 were treated with one dose of 5 mg/kg infliximab (Table 1). In all 11 cases, ICIs were subsequently discontinued permanently. The median follow-up time from the onset of ICI myocarditis was 279 days in the non-infliximab group and 287 days in the infliximab group. As expected, patients treated with infliximab had more severe disease by the ASCO classification compared to those not treated with infliximab. All four patients requiring treatment with infliximab had grade 4 myocarditis and experienced a MACE prompting infliximab initiation. Aside from age, there were no significant differences in baseline patient characteristics between patients treated with and without infliximab including the type of cancer and type of ICI therapy. Patients treated with infliximab were younger (72.7 vs. 61.8 years, *p* = 0.02). Notably there was no significant difference in the total number of ICI doses received (2.0 vs. 2.5, *p* = 0.77) or duration from initial ICI dose to onset of symptoms (53 vs. 75 days, *p* = 0.71) between the two groups. There was a nonsignificant trend toward higher admission and peak troponins (0.7 vs. 0.2 ng/ml *p* = 0.089 and 1.0 vs. 0.2 ng/mL *p* = 0.13, respectively) in the infliximab group. The time to troponin normalization was numerically longer in the infliximab group (151.5 vs. 58 days *p* = 0.25). Both groups had pre-ICI ejection fractions > 50 % and both groups had moderately reduced ejection fractions at diagnosis of ICI myocarditis (42.5 % vs. 38.8 % *p* = 0.73). The duration of prednisone taper was numerically longer in the infliximab group (90 vs. 150 days *p* = 0.32). All patients survived initial hospital admission for ICI-associated myocarditis.

**Table 1:** Baseline characteristics comparing patients with immune checkpoint inhibitor myocarditis treated with and without infliximab

	Not Treated with Infliximab	Treated with Infliximab	*p*-value
	*N* = 7	*N* = 4	
**Baseline Patient Characteristics**			
Age, mean (SD)	72.7 (6.5)	61.8 (4.6)	0.016
Gender (male)	6 (86 %)	2 (50 %)	0.20
BMI, mean (SD)	23.4 (4.8)	27.7 (9.8)	0.35
Hypertension	2 (29 %)	2 (50 %)	0.48
Diabetes	1 (14 %)	0 (0 %)	0.43
Tobacco Use	0 (0 %)	0 (0 %)	
Coronary Artery Disease	1 (17 %)	0 (0 %)	0.39
Heart Failure	2 (29 %)	0 (0 %)	0.24
Cerebral Vascular Accident	7 (100 %)	4 (100 %)	
Obstructive Sleep Apnea	7 (100 %)	4 (100 %)	
Chronic Kidney Disease	2 (29 %)	1 (25 %)	0.90
**Baseline Cancer Demographics**			
Total No. of ICI doses, median (IQR)	2.0 (1.0, 14.0)	2.5 (1.5, 6.0)	0.77
Time from ICI dose to Onset of Symptoms, Days, (median IQR)	53.0 (21.0, 424.0)	74.0 (52.0, 171.5)	0.71
Malignancy			0.23
Metastatic Melanoma	1 (14 %)	2 (50 %)	
Metastatic RCC	0 (0 %)	1 (25 %)	
Ovarian Adenocarcinoma	0 (0 %)	1 (25 %)	
NSCLC	3 (43 %)	0 (0 %)	
Metastatic SCC of tongue	1 (14 %)	0 (0 %)	
Laryngeal SCC	1 (14 %)	0 (0 %)	
DLBCL	1 (14 %)	0 (0 %)	
Immune Checkpoint Inhibitor			0.31
Nivolumab	2 (29 %)	3 (75 %)	
Pembrolizumab	4 (57 %)	1 (25 %)	
Darvalumab	1 (14 %)	0 (0 %)	
Combined ICI (anti-CTLA-4 + anti-PD1/PDL1)	1(14 %)	0 (0 %)	0.77
Prior Chemotherapy or Radiation			
Radiation	6 (86 %)	1 (25 %)	0.044
Anthracycline	0 (0 %)	0 (0 %)	
VEGF inhibitors	0 (0 %)	0 (0 %)	
**Myocarditis Presentation and Management**			
Follow up time, days (mean SD)	279.0 (219.8)	287.2 (258.2)	0.96
BNP, mean (SD)	8562.0 (14856.4)	11749.3 (9355.2)	0.75
Admission Troponin, ng/ml (median IQR)	0.2 (0.0, 0.6)	0.7 (0.4, 4.8)	0.089
Peak Troponin ng/ml (median IQR)	0.2 (0.1, 0.6)	1.0 (0.5, 4.9)	0.13
Time for Troponin Normalization, days (median IQR)	1740 (1245, 2160)	4545 (1680, 9315)	0.25
ICI Myocarditis Grade			0.023
1	0 (0 %)	0 (0 %)	
2	4 (57 %)	0 (0 %)	
3	2 (29 %)	0 (0 %)	
4	1 (14 %)	4 (100 %)	
Clinical Presentation			
MACE	1 (14 %)	4 (100 %)	0.006
Congestive Heart Failure	4 (57 %)	4 (100 %)	0.12
Cardiogenic Shock	0 (0 %)	2 (50 %)	0.039
Complete Heart Block	1 (14 %)	2 (50 %)	0.20
Ventricular Tachycardia	0 (0 %)	4 (100 %)	< 0.001
Cardiac Arrest	1 (14 %)	1 (25 %)	0.66
**Echocardiogram**			
Pre-ICI EF, mean (SD) (*n* = 4)	57.5 (15.0)	63.3 (2.9)	0.54
New EF, mean (SD)	42.5 (20.2)	38.8 (4.8)	0.73
Mitral Inflow E, cm/s (mean SD) (*n* = 7)	85 (27)	75 (31)	0.65
Mitral Inflow A, cm/s (mean SD) (*n* = 7)	89 (13)	50 (9)	0.007
E/A, mean (SD) (*n* = 7)	0.9 (0.3)	1.5 (0.7)	0.16
Average Mitral E/e’, (mean SD) (*n* = 7)	12.3 (3.0)	12.3 (7.5)	0.99
**Cardiac MRI**			
ECV, % (*n* = 4)	32.0	35.5	0.65
Native T1 value (ms) (*n* = 5)	1076 (102)	1130 (27)	0.53
Predominant LGE Pattern (*n* = 6)			0.63
Sub-endocardial/Transmural	0	2	
Sub-epicardial	0	0	
Mid-myocardial	2	2	
Diffuse	0	0	
Treatment			
Initial Treatment on Presentation	Prednisone 1 mg/kg	IV solumedrol 1 g x3 days and infliximab 5 mg/kg	
Prednisone Duration, days (median IQR)	90 (60,150)	150 (75, 300)	0.32
Survival at discharge	7 (100 %)	4 (100 %)	

Abbreviations: BMI, body mass index; BNP, B-type natriuretic peptide; CTLA-4, cytotoxic T-lymphocyte associated protein 4; extracellular volume, ECV; DLBCL, diffuse large B-cell lymphoma; late gadolinium enhancement, LGE; EF, ejection fraction; MACE, major adverse cardiovascular ev ent; NSCLC, non-small cell lung cancer; No., number; PD-L1, programmed death-ligand 1; ICI, immune checkpoint inhibitor; RCC, renal cell carcinoma; SCC, squamous cell cancer; VEGF, vascular endothelial growth factor

**Fig. 1:**
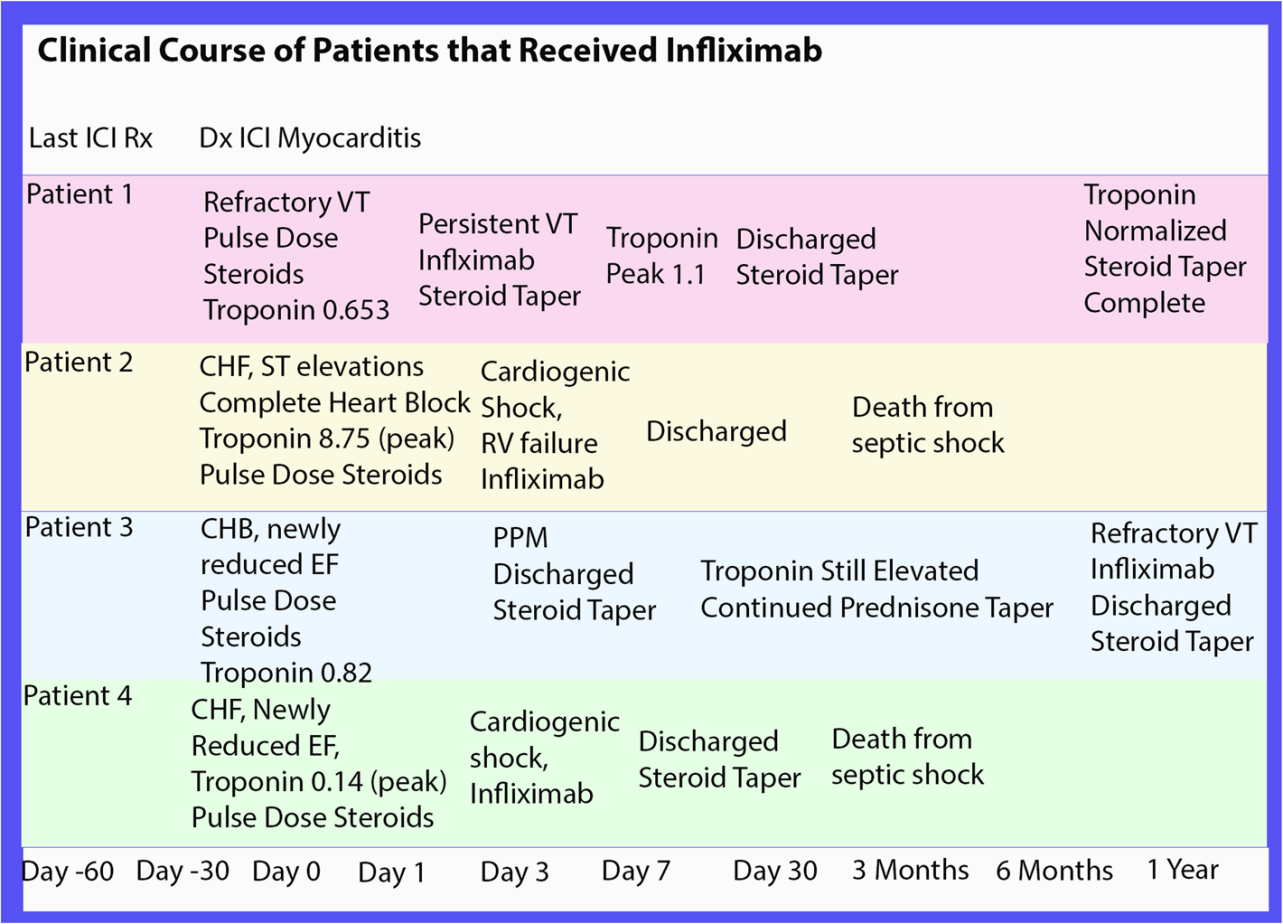
Clinical Course of Patients that Received Infliximab. The clinical course of ICI myocarditis patients treated with infliximab over time. Abbreviations: CHB, complete heart block; CHF, congestive heart failure; Dx, Diagnosis; EF; ejection fraction, ICI, immune checkpoint inhibitory; PPM, permanent pacemaker; Rx, Treatment; RV, right ventricle; VT, ventricular tachycardia

All four patients who received infliximab were admitted to the cardiac intensive care unit. Patient 1 presented with refractory ventricular tachycardia (VT) despite anti-arrhythmic therapy and pulse dose steroids (Fig. 1). Patient 2 presented with severe right heart failure (RHF) with subsequent cardiogenic shock, refractory VT and complete heart block (CHB). Patient 3 initially presented with a newly reduced EF and hemodynamically unstable CHB requiring a permanent pacemaker (PPM). This patient required a prolonged steroid taper due to persistent troponin elevations and during the 8th month of this taper, he was readmitted for refractory VT. Patient 4 presented with inotrope-dependent cardiogenic shock. Two patients received infliximab (infliximab 5 mg/kg) after completing three doses of 1 g of intravenous solumedrol and two patients received infliximab after the first dose of intravenous solumedrol. All 4 patients received infliximab due to worsening clinical status despite the high-dose steroids. All 4 patients survived initial hospitalization but required prolonged steroid tapers guided by serial troponin monitoring. Over a median follow-up period of 287 days, two of the 4 patients died from septic shock 2 and 3 months after initial treatment of their myocarditis; one of these patients was on a steroid taper and the other patient had just completed a steroid taper. The other two patients have completed their steroid tapers without further evidence of myocarditis at their most recent follow-up.

## Discussion

Glucocorticoids remain the cornerstone of treatment for ICI myocarditis. Most patients experienced clinical improvement and biomarker normalization with steroid monotherapy. However, there remain several gaps in our understanding of the optimal medical therapy. There is no consensus on the initial dosing of steroids, the type of steroid, addition of alternative immunosuppressive agents when necessary, or outpatient steroid tapering protocols.

Mahmood et al. observed that MACE occurred less frequently in patients who received high-dose (> 2 mg/kg of prednisone or equivalent) steroids compared with those receiving low-dose steroids (< 1 mg/kg of prednisone or equivalent) [7]. In addition, Zhang et al. showed that initiating corticosteroids earlier and at higher doses was associated with lower risk of MACE [15]. The ASCO guidelines recommend 1 mg/kg of prednisone or equivalent either by mouth or intravenously [13]. Our clinical practice has been to administer 1 gram of intravenous solumedrol daily for three days, followed by a 1–2 mg/kg steroid taper of oral prednisone. The ASCO clinical practice guidelines for irAEs recommend a glucocorticoid taper over 4–6 weeks while trials for viral myocarditis have examined steroid treatment durations of at least 3 months and in some cases up to a year [12]. For ICI-mediated myocarditis, most case reports and case series track response to steroids by monitoring troponin levels. If the troponin levels increase, our practice has been to increase the steroid dose and extend the taper. This situation arose in one patient in our cohort who had a significant clinical deterioration when the troponin levels began to rise despite steroid therapy. However, we acknowledge that there is a lack of research into a standardized steroid protocol in these patients. Additionally, whether the ICI should be permanently discontinued or whether restarting ICI therapy is safe remains controversial. Our institution has not restarted anyone on ICI therapy after the development of grade 4 myocarditis. In addition to immunosuppressive therapy, patients should also receive appropriate cardiac support including inotropes, mechanical circulatory support, and management of arrhythmias including temporary pacemakers and external defibrillators when indicated. Our approach has been to treat ICI myocarditis aggressively as a potentially reversible complication of therapy even in patients with advanced incurable cancer. Decisions regarding levels of mechanical circulatory support offered to patients with active malignancy but a reversible cause of cardiac decompensation are controversial and need to be discussed with heart failure, cardiothoracic surgery and oncology. The intensity of therapy offered to individual patients should always be consistent with their stated goals of care.

There have been case reports or small case series describing the use of several immunomodulating agents for ICI-induced myocarditis with varying degrees of efficacy. These therapies include intravenous immunoglobulin, [16, 17] mycophenolate, [17] anti-thymocyte globulin, [18] plasmapheresis, [17] infliximab, [17] alemtuzumab, [19]] and abatacept [20]. In our cohort, we have demonstrated that infliximab, a chimeric IgG1 monoclonal antibody that blocks tumor necrosis factor-alpha, appears to be an effective and safe agent for steroid-refractory ICI myocarditis. Although there is a black box warning regarding the potential for infliximab to worsen heart failure based on observations by Kwon et al. of the development of heart failure in patients with rheumatoid arthritis treated with infliximab, [21] we have demonstrated in a small sample that infliximab safely improved decompensated heart failure and cardiogenic shock due to ICI myocarditis. In our limited experience, a single dose of infliximab 5 mg/kg has been effective and safe. While we have not re-dosed infliximab at our institution, there have been case series describing additional 5 mg/kg doses or a single dose of 10 mg/kg [22, 23]. There are limited data outside oncology suggesting that 5 mg/kg may be safer in treating patients with heart failure not due to ICIs [24].

In the setting of therapeutic immunosuppression with both infliximab and prolonged high dose steroids, patients are at increased risk for opportunistic infections and need to be monitored closely. Two of the four patients in our series eventually died of septic shock, so it is formally possible that these events were due to the effects of combined immune suppression with infliximab and glucocorticoids. Anti-TNF agents such as infliximab have been associated with an increased risk of mycobacterial infections, invasive fungal infections, viral infections as well as bacterial pathogens [25–27]. Prior to starting therapy, patients should be screened for HIV and Hepatitis B and C, a history of invasive fungal infections and should undergo assessment for active or latent tuberculosis. Concern for any of these infections warrants consultation with Infectious Diseases. Of note, immediate therapy for ICI myocarditis may be necessary and should not be withheld even with infectious screening tests pending, particularly if the patient is in cardiogenic shock or having sustained life-threatening arrythmias refractory to other therapies.

To prevent infections, we recommend patients receive prophylaxis for *Pneumocystis jirovecii* pneumonia until the steroid taper dose is below 20 mg of prednisone or equivalent, ideally with trimethoprim-sulfamethoxazole. There are insufficient data at this time to recommend prophylaxis against invasive fungal infections [27, 28]. Patients should also be up to date on all appropriate vaccinations, noting that administration may be deferred in the acute setting given significant immunosuppression and lower likelihood of vaccine response.

**Fig. 2:**
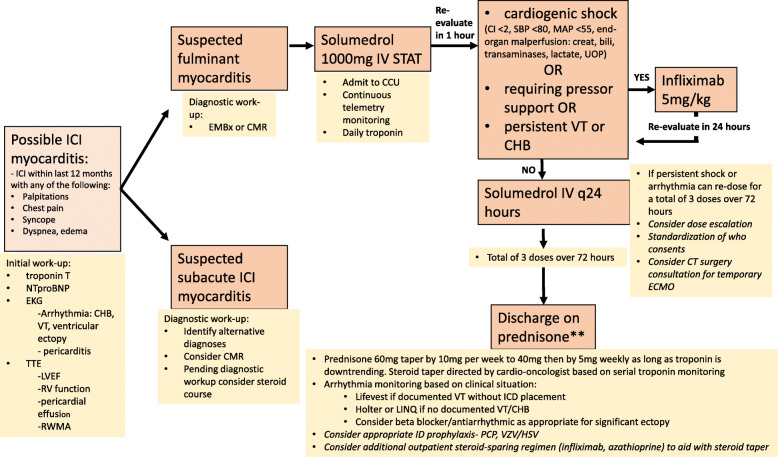
Suggested Treatment Algorithm for Suspected Immune Checkpoint Inhibitor Myocarditis. Abbreviations: CHB, complete heart block; CI, cardiac index; CMR, cardiac MRI; EMBx, endomycardial biopsy; HSV, herpes simplex virus; ICD, internal cardiac defibrillator; IV, intravenous; PCP, pneumocystis pneumonia; RWMA, regional wall motion abnormality; TTE, transthoracic echocardiogram; VT, ventricular tachycardia, VZV; varicella zoster virus

While we have only used infliximab in steroid-refractory cases, further study should consider earlier initiation of infliximab or other additional immunosuppressive agents together with initial high-dose steroids in patients with grade 3 or 4 myocarditis. Early initiation of a second immunosuppressive agent such as infliximab may reduce the total duration of steroid exposure. Given the potential effect of corticosteroids on T-cell function, prolonged use in patients with advanced cancer may decrease the efficacy of ICIs and is generally avoided whenever possible [29, 30]. Additionally, initial dosing of steroids should be studied further; data from heart transplant cellular rejection, which has similarities to T-cell driven ICI myocarditis, has shown equivalent outcomes with 500 mg and 1000 mg of intravenous solumedrol [31, 32]. While many questions remain unanswered and additional prospective studies are needed to address the best ways to manage ICI-related cardiovascular adverse events, we believe it is prudent to have a multidisciplinary team to help manage these high-risk patients. The Fig. 2 is a suggested algorithm for the treatment of ICI myocarditis developed by a multi-disciplinary team involving medical oncology, heart failure, cardiothoracic surgery, cardio-oncology, and pharmacists. We recognize that this protocol will surely change as more research is done within this field, but we hope this serves as a guide until then as there is currently a paucity of data and treatment recommendations for ICI myocarditis.

There are several limitations to the current study. This is a single center study, which limits its generalizability. This study is also limited by its retrospective nature, which increases the possibility of comorbidity misclassification. The lack of control subjects makes it difficult for comparative evaluation to determine if the standard therapy also produced the same outcomes, although it may be difficult to power studies in this population for clinically important outcomes. Given the small sample size, our study has insufficient power to detect small changes in the effectiveness and safety associated with infliximab. A larger sample size is needed to confirm our findings.

## Conclusions

ICI-related myocarditis is an uncommon entity, making it difficult to study in randomized trials. Guidance for its management is therefore based on small case series and case reports. Current treatment focuses on glucocorticoids with a possible role for more targeted immune modulators in patients with severe disease or disease that responds poorly to steroids. We have demonstrated efficacy and safety of infliximab in patients with severe presentations of ICI myocarditis. Use of infliximab did not worsen heart failure in any case despite the black box warning for infliximab in patients with heart failure. With the variable presentation of ICI myocarditis and the availability of various immunosuppressive agents and strategies, it is prudent to involve a multi-disciplinary team to optimize management of these complex patients. As the indications and use for ICI continue to expand, the incidence and recognition of ICI myocarditis will likely increase. Therefore, further additional studies are indicated to help guide treatment of this complication of cancer immunotherapy.

## Data Availability

All data generated or analysed during this study are included in this published article.

## Abbreviations

ASCO: American Society of Clinical Oncology
CHB: Complete heart block
MACE: Major adverse cardiovascular events
ICI: Immune checkpoint inhibitor
irAE: Immune-related adverse events
VT: Ventricular Tachycardia
